# On the Use of Oblique Acoustic Leakage to Measure the Wavenumber Spectrum of Propagating Lamb Waves

**DOI:** 10.3390/s19061391

**Published:** 2019-03-21

**Authors:** Christopher Hakoda, Mostafa Hasanian, Cliff J. Lissenden

**Affiliations:** Department of Engineering Science and Mechanics, Pennsylvania State University, University Park, PA 16802, USA; mzh209@psu.edu (M.H.); Lissenden@psu.edu (C.J.L.)

**Keywords:** air-coupled transducer, Lamb waves, 2DFFT, wavenumber spectrum, Snell’s law, slowness curves

## Abstract

Lamb waves propagating within a waveguide often have similar frequency content. If there are multiple Lamb wave modes with the same frequency content, the wavenumber spectrum can be used to distinguish between them. As a result, the wavenumber spectrum is an important tool for determining the modal content of signals. In this paper, we propose a new method for measuring wavenumber spectra that uses an air-coupled transducer for reception and Snell’s law instead of a fast-Fourier transform. The method employs an angular scan rather than a translational scan. The advantages and disadvantages of the method are discussed along with some suggestions for potential improvements. Finally, experimental results comparing the proposed method to a more conventional method, which used a PVDF transducer, demonstrate the feasibility of the proposed method.

## 1. Introduction

Acoustic leakage from elastodynamic waveguides into fluid half-spaces has been studied extensively through a number of theoretical methods (e.g., [1,2,3]). It is known that the acoustic leakage from a plate waveguide will take the form of an oblique pressure wave or an evanescent pressure wave, as plotted by Hayashi and Inoue [1] and measured in great detail by Courouble and Moufle [4]. Acoustic leakage is a well understood phenomenon and is known to follow Snell’s law for excitation and reception [5,6,7,8,9]. That is, it is conventionally used to determine the optimum angular position for an air-coupled transducer, whether it is for excitation or reception. In fact, the necessity of accurate angular positioning was highlighted by Castaings et al. [9] who observed that the amplitude of the measured signal could change significantly for less than one degree of misalignment. Alternatively, some analysts sweep through angular positions to find an optimum angle, as was done by Baldwin et al. [10].

In the context of elastodynamic guided waves, a wavenumber spectrum is used to determine the modal content of waves propagating in a waveguide. It is often calculated in conjunction with a frequency spectrum by computing a two-dimensional fast Fourier transform (2DFFT) from A-scans measured at evenly spaced intervals along the surface of the waveguide [11]. This method of analysis has been used with great success in the field of structural health monitoring and non-destructive evaluation (e.g., [12,13,14,15,16,17]). Implementations focused on non-contact measurements sometimes use air-coupled transducers to measure the A-scans required to calculate the 2DFFT. The air-coupled transducer is usually oriented at an angular position dictated by Snell’s law and then it is translated parallel to the surface of the waveguide to record A-scans at evenly spaced intervals [17,18,19,20,21].

In this study, the angular dependence of the acoustic leakage was used to measure the wavenumber spectrum of Lamb waves travelling in an isotropic plate waveguide. The proposed method relies on Snell’s law, as opposed to the conventional fast-Fourier transform, to measure the wavenumber spectrum. Although this is reasonably simple, the authors could not find a publication where this was investigated. The intent of the current investigation was to provide analysts with another method for measuring the wavenumber spectrum of an elastodynamic guided wave and to emphasize the importance of angular positioning when using an air-coupled transducer.

Section 3 covers the basic theory related to slowness curves and how they can be exploited as a means to interpret the conversion from different angular orientations to a wavenumber spectrum. A potential approach to re-scaling mode-dependent acoustic leakage is also proposed in this section. Section 4 describes the experimental setup that was used to compare the proposed method to a conventional 2DFFT approach. Section 5 then discusses the results of the experiment.

## 2. Theory and Concept

The method for measuring the wavenumber spectrum that is detailed in this paper relies upon Snell’s law for refraction at an interface between distinct media 1 and 2,

(1) $$\frac{\sin\left(\theta_{1}\right)}{c_{1}}=\frac{\sin\left(\theta_{2}\right)}{c_{2}}$$

and is assisted by slowness curves. That is, it is possible to show that Lamb waves typically emit acoustic leakage in the form of an oblique pressure wave. The angle of incidence of the pressure wave corresponds with that Lamb wave’s wavenumber. Thus, by measuring the pressure wave at a variety of angles, a wavenumber spectrum for the Lamb waves in the waveguide can be obtained.

### 2.1. General Theory and Concept

Beginning from balance laws, the expression for slowness curves of an elastic solid can be calculated using the balance of linear momentum in the absence of body forces,

(2) $$\sigma_{ij,j}=\rho {\ddot{u}}_{i}.$$

Hooke’s Law,

(3) $$\sigma_{ij}=C_{ijkl}\epsilon_{kl},$$

and the linearized strain–displacement relationship,

(4) $$\epsilon_{kl}=\frac{1}{2}\left(u_{k,l}+u_{l,k}\right).$$

Next, we assume plane wave propagation,

(5) $$u_{i}\left(x,y,z,t\right)=U_{i}e^{-ik_{j}r_{j}+i\omega t},$$

to derive the Christoffel equation,

(6) $$C_{ijkl}k_{j}k_{k}U_{i}-\rho \omega^{2}U_{i}=0,$$

and restrict the wave vector to the x-z plane (i.e., $k_{y}=0$). Assuming isotropic material properties, arranging the equations into matrix form,

(7) $$A^{\left(solid\right)}U_{i}=0,$$

and solving for the non-trivial solutions,

(8) $$\det\left(A^{\left(solid\right)}\right)=0,$$

gives the expression for the slowness curves of an isotropic elastic solid,

(9) $$\frac{k_{x}^{2}}{\omega^{2}}+\frac{k_{z}^{2}}{\omega^{2}}=\frac{1}{c_{L}^{2}},$$

(10) $$\frac{k_{x}^{2}}{\omega^{2}}+\frac{k_{z}^{2}}{\omega^{2}}=\frac{1}{c_{S}^{2}}.$$

Likewise, the slowness curve of a fluid can be calculated by using the linearized acoustic equations [22] that consist of the state equation,

(11) $$\rho^{\prime }=\frac{p^{\prime }}{c_{air}^{2}},$$

mass balance,

(12) $$\dot{\rho^{\prime }}+\rho_{o}v_{i,i}^{\prime }=0,$$

and balance of linear momentum,

(13) $$\rho_{0}{\dot{v}}_{i}^{\prime }=-p_{,i}^{\prime }.$$

By taking the gradient of a combination of the state equation and the mass balance, and substituting the time-derivative of the balance of linear momentum into this, the following governing equation can be derived,

(14) $$v_{i,ij}^{\prime }-\frac{1}{c_{air}^{2}}{\ddot{v}}_{j}^{\prime }=0.$$

Furthermore, by assuming the same plane wave solution (Equation (5)), the governing equation becomes,

(15) $$U_{i}^{\prime }k_{i}k_{j}-\frac{\omega^{2}}{c_{air}^{2}}U_{j}^{\prime }=0.$$

Restricting the wavevector to the x-z plane (i.e., $k_{y}=0$), assuming quiescence [22], and organizing the equations into a matrix form,

(16) $$A^{\left(air\right)}U_{j}=0,$$

non-trivial solution can be solved for,

(17) $$\det\left(A^{\left(air\right)}\right)=0,$$

which gives the slowness curve for air,

(18) $$\frac{k_{x}^{2}}{\omega^{2}}+\frac{k_{z}^{2}}{\omega^{2}}=\frac{1}{c_{air}^{2}}.$$

**Figure 1:**
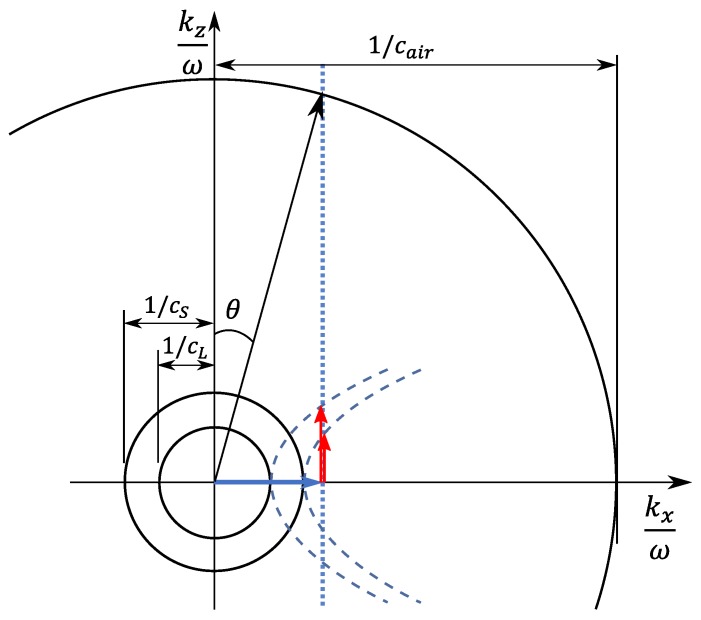
Schematic (not to scale) of how the slowness curves and Snell’s law can be used to characterize the direction in which the leaking wave (oblique pressure wave) is travelling. The black circles represent the bulk wave solutions (real-valued $k_{z}$) and the blue parabolas represent the evanescent wave solutions (imaginary-valued $k_{z}$).

In the context of this paper, the importance of slowness curves is based on how well they work with Snell’s law [23,24]. That is, Snell’s law still applies at the solid–fluid interface due to the few remaining continuity conditions. By using Snell’s law to join the slowness curves of the fluid and solid, the wavevector of a wave travelling in the solid can be quantitatively related to the wavevector of a wave travelling in the fluid. For the remainder of the paper, it is assumed that the wave in the solid media is travelling in the x-direction. The slowness curves for the fluid and solid are illustrated on the same slowness axes in Figure 1. Points of intersection between the vertical dashed-line and the slowness curves are indicative of Christoffel solutions that are consistent with Snell’s law.

**Figure 2:**
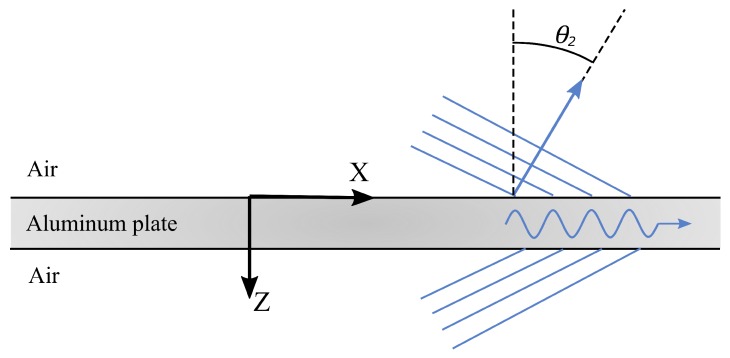
Schematic of the plate waveguide being investigated.

For the case of a Lamb wave travelling within an aluminum plate (as shown in Figure 2), where the plate’s surface is normal to the z-axis, Figure 1 still applies because Lamb waves are representable as a superposition of the Christoffel equation solutions [25]. In this context, the phase velocity of the Lamb wave is equal to $\omega /k_{x}$. This makes Figure 1 usable as a simple tool for predicting acoustic leakage characteristics. Of these acoustic leakage characteristics, this paper focuses on two of them:

- Due to the large difference between the wave speeds in air and metals, the acoustic leakage from Lamb waves is in the form of obliquely-travelling bulk waves (except for the A0 mode at low frequencies that have a phase velocity less than $c_{air}$).
- As a result of Snell’s law, each angle of incidence of the obliquely-travelling acoustic leakage corresponds with the $k_{x}$ value of the waves travelling within the solid. This $k_{x}$ is equal to the wavenumber of the Lamb waves travelling in the x-direction.

Since the angle of incidence of the acoustic leakage corresponds with the wavenumber of Lamb waves travelling in the plate, by taking measurements with an air-coupled transducer that is positioned at various angles, a wavenumber spectrum can be measured without using a fast-Fourier transform (FFT). That is, each angular position of the air-coupled transducer is equated to a wavenumber by using Snell’s law,

(19) $$k_{x}=\frac{\omega \sin\left(\theta_{2}\right)}{c_{air}}.$$

The air-coupled transducer is hindered by the fact that various Lamb waves do not emit the same amount of acoustic leakage. The following subsection seeks to theoretically quantify this difference in acoustic leakage, while proposing a potential method to correct for it.

### 2.2. Adjusting for Mode-Dependent Acoustic Leakage

To quantify the difference in acoustic leakage between Lamb wave modes, the ratio of the acoustic leakage amplitude to the wave-structure (i.e., displacement profile through the cross-section of the waveguide) amplitude is used as a parameter. To calculate the amplitude of the acoustic leakage, the partial wave method is used [26] and it is assumed that the dispersion curves of the traction-free plate waveguide are a good approximation of the plate waveguide surrounded by air. This assumption is common for experimentalists who use ultrasonic guided waves for structural health monitoring or non-destructive testing. The following interface conditions are used to calculate the wave-structures of the air-coupled Lamb waves,

(20) $$\begin{array}{cc} p^{\prime } & =-\sigma_{zz}\;\mathrm{at}\;z=0,\\ u_{z}{|}_{air} & =u_{z}{|}_{aluminum}\;\mathrm{at}\;z=0,\\ \sigma_{xz} & =0\;\mathrm{at}\;z=0,\\ p^{\prime } & =-\sigma_{zz}\;\mathrm{at}\;z=H,\\ u_{z}{|}_{air} & =u_{z}{|}_{aluminum}\;\mathrm{at}\;z=H,\\ \sigma_{xz} & =0\;\mathrm{at}\;z=H, \end{array}$$

where in the air,

(21) $$u_{i}\left(x,z,t\right)=B^{\left(air\right)}{\hat{k}}_{i}e^{-ik_{j}r_{j}+i\omega t},$$

(22) $$p^{\prime }\left(x,z,t\right)=iB^{\left(air\right)}\rho_{o}c_{air}\omega e^{-ik_{j}r_{j}+i\omega t}.$$

Equation (21) assumes that the particle polarization is parallel to the wave propagation direction. Equation (22) uses Equation (13) and the plane wave assumption to calculate an expression for the pressure.

Once the amplitude of the acoustic leakage is calculated, it is normalized according to the magnitude of the complex-valued wave-structure at the air–plate interface,

(23) $$m:=\frac{|B^{\left(air\right)}|}{\sqrt{|u_{x}{\left(z=0\right)|}^{2}+{\left|u_{z}\left(z=0\right)\right|}^{2}}}.$$

**Figure 3:**
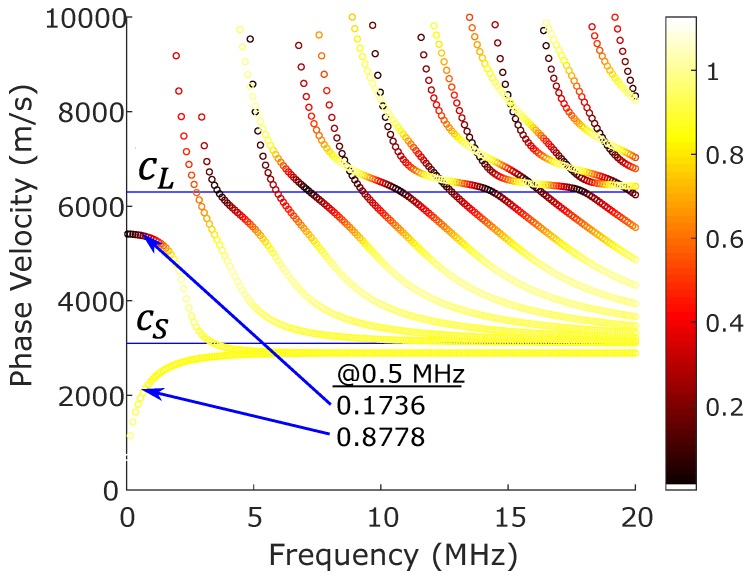
Normalized acoustic leakage, m, is represented as a color scale and overlaid on the dispersion curves for a 1-mm thick aluminum plate. Aluminum properties: $c_{l}=6350$ m/s, $c_{S}=3100$ m/s and ρ=2700 kg/m^3^.

This parameter is a ratio between the magnitude of the acoustic leakage’s displacement, $|B^{\left(air\right)}|$, and the magnitude of the Lamb wave displacement at the interface. The displacement at the surface was chosen as a representative characteristic of the Lamb wave since Lamb waves are conventionally measured at the surface of the plate waveguide. An energy density ratio or a pressure–stress ratio was not chosen since the large difference in material properties makes the numerical comparison difficult. Figure 3 shows the parameter, *m*, as a color variable that is overlaid on the dispersion curves for a 1-mm thick aluminum traction-free plate waveguide.

To correct for this difference in acoustic leakage, a mode-dependent linear scaling that uses the *m* parameter is proposed. That is, considering the relative amount of acoustic leakage, *m*, for each mode at a given center frequency, the measured spectrum should be re-scaled to provide a more accurate representation of the waves propagating within the plate.

## 3. Experimental Setup

**Figure 4:**
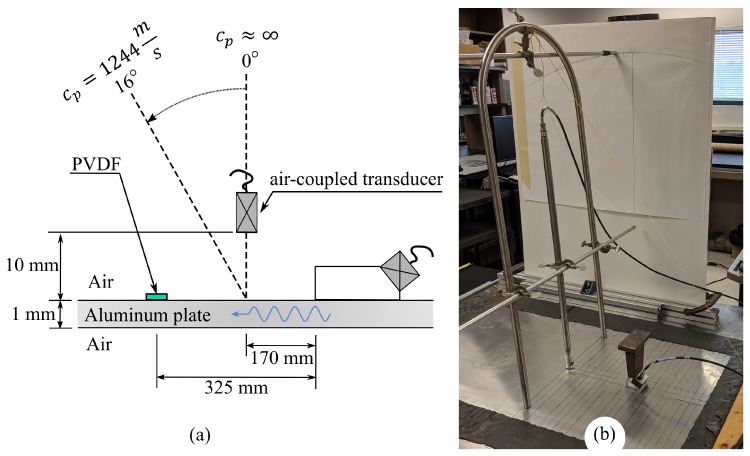
(**a**) Not-to-scale schematic of the experimental setup; and (**b**) picture of the angular positioning frame for air-coupled transducer measurements. The angle wedge transducer for excitation is also shown with dead weight to prevent movement.

An experiment was designed to verify the effectiveness of using the angular positioning of an air-coupled transducer to measure the wavenumber spectrum of Lamb waves. A five-cycle pulse with a center frequency of 0.5 MHz was used with a 30° Plexiglas angle-wedge transducer to preferentially excite the S0 Lamb mode, and some A0 Lamb mode, within a 1-mm-thick aluminum plate waveguide. Only the A0 and S0 fundamental modes could be generated at this frequency. These Lamb waves were measured using two transducers, a Polyvinylidene difluoride (PVDF) transducer that is 1 mm × 30 mm in area and a 0.5 MHz air-coupled transducer. The PVDF transducer was used to measure a conventional frequency-wavenumber spectrum by calculating the 2DFFT of 31 A-scans measured in 1-mm increments. The air-coupled transducer was attached to a frame that pivots at the surface of the plate while the wedge transducer was kept in place, as shown in Figure 4. By measuring the angle at a radial distance of about 0.9 m from the pivot point, the angular position was changed in 15-min increments. The range of the angular position was from 0° to 16° as this was deemed sufficient for measuring both the S0 and A0 modes at 0.5 MHz, which should occur at about 3.7° and 10.2° (i.e., 5315 m/s and 1937 m/s), respectively. At 0°, the air-coupled transducer was positioned with a liftoff of 1 cm from the plate waveguide’s surface; if the transducer were too close to the plate, then an angular re-positioning would cause the transducer’s rim and the plate to touch.

The 2DFFT results from the PVDF transducer were treated as an accurate representation (i.e., the baseline) of the Lamb wave spectra within the plate, while the spectra from the air-coupled transducer were evaluated on how well they agreed with the baseline. The PVDF transducer was chosen as the accurate representation because of its wide bandwidth of reception and its sensitivity to both in-plane and out-of-plane displacement according to its $d_{31}$ and $d_{33}$ piezoelectric strain coefficients [27].

## 4. Experimental Results

**Figure 5:**
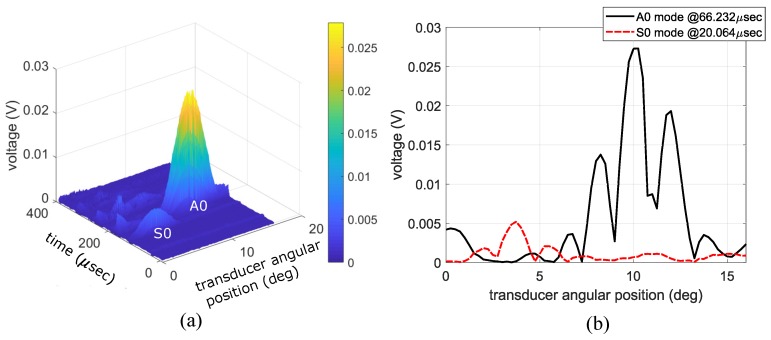
(**a**) Surface plot of the absolute value of the A-scan with respect to the angular positioning of the receiving air-coupled transducer; and (**b**) slices of the surface plot in (**a**) at specific times to highlight and compare the peaks of the S0 and A0 modes.

**Figure 6:**
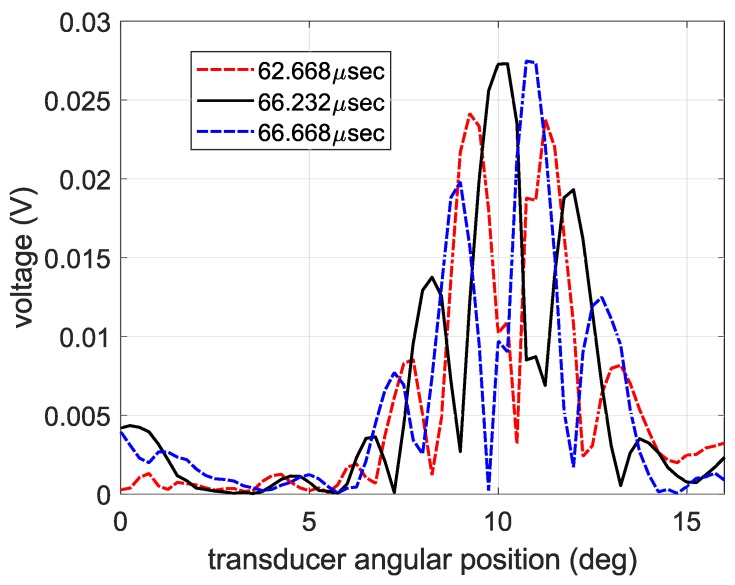
Three slices of the surface plot in Figure 5a taken at the beginning, middle and end of the A0 waveform.

In agreement with the literature [9,28], it became clear when collecting data that there was a significant variation in signal strength depending on the angular positioning of the receiving air-coupled transducer. Figure 5 shows how great this variation can be and that the two main features are clearly dependent on the angular position and time-of-flight. The apparent disagreement between Figure 5a,b is caused by the low directivity of the air-coupled transducer. This introduced a blurring effect when plotted, as in Figure 5a, and is believed to be partially caused by changes in time-of-flight when measuring the acoustic leakage off-axis from the transducer face. This is highlighted in Figure 6, where multiple slices of the surface plot at different times in Figure 5a are plotted. The slices correspond with the beginning, middle, and end of the A0 waveform. When combined, these slices resemble the A0 peak seen in Figure 5a.

**Figure 7:**
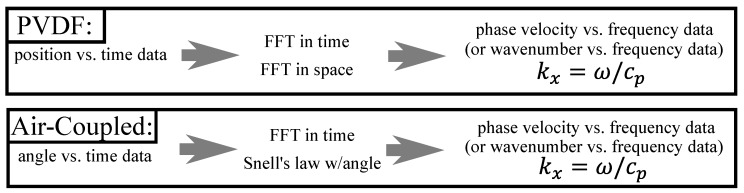
Flow chart of the methods used to calculate the wavenumber–frequency spectra of the PVDF and air-coupled transducer data.

**Figure 8:**
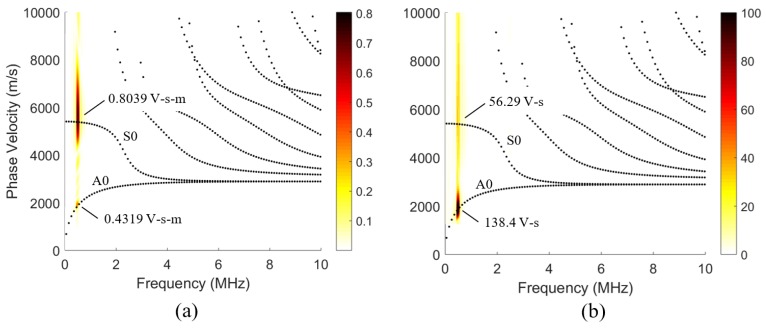
Phase velocity-frequency spectrum from: (**a**) the PVDF transducer; and (**b**) the air-coupled transducer when used to receive a 0.5 MHz pulse from a Plexiglas-wedge transducer with a 30° angle of incidence. Color axis restricted for easier viewing of features. Numerically calculated Lamb wave dispersion curves (black dots) added for reference.

**Figure 9:**
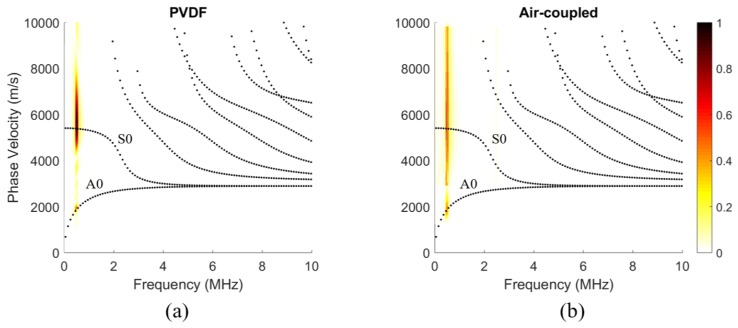
Lamb wave dispersion curves with: (**a**) a normalized phase velocity–frequency spectrum that was measured using the PVDF transducer; and (**b**) s re-scaled, then normalized phase velocity–frequency spectrum measured using the air-coupled transducer. Numerically calculated Lamb wave dispersion curves (black dots) added for reference.

The spectra in the following subsections were calculated using a 2DFFT, as shown by the flow chart in Figure 7. Most notably, Snell’s law replaced the conventional spatial FFT when calculating the spectra of the air-coupled transducer data. That is, the spectra of the PVDF data were calculated using a 2DFFT in space and time, while the spectra of the air-coupled data were calculated using an FFT in time and Snell’s law to find the wavenumber spectrum. The spectra calculated from the PVDF data are shown in Figure 8a and reveal that a strong S0 Lamb wave is propagating within the plate, along with a small amount of A0 Lamb wave, as expected for 0.5 MHz excitation on a 30° wedge. The spectra in Figure 8 and Figure 9 are overlaid on numerically calculated Lamb wave dispersion curves for reference. The spectra from the air-coupled data are shown in Figure 8b and indicate a large amount of A0 Lamb wave propagating within the plate. The maximum values of the spectra in the S0 and A0 regions are labeled, thereby highlighting the fact that the color axes have significantly different values due to their different units. That is, the air-coupled spectra did not require an FFT for the wavenumber spectrum since each angular position corresponds with a wavenumber value. Although difficult to see in Figure 8b, there was a lower limit to the range of phase velocities that could be measured, and the resolution of the spectra decreased for larger phase velocities. The lower limit existed because we did not measure A-scans past an angular position of 16° and the decreased resolution at larger phase velocities was due to the phase velocity being inversely related to the sine of the angular position. That is, smaller increments and higher accuracy in angular position within the 0° to 7° range would yield better resolution.

The seemingly contradictory results in Figure 8a,b was due to the acoustic leakage’s aforementioned mode dependence shown in Figure 3. Based on the wave-structure, the S0 mode was expected to emit significantly less acoustic leakage than the A0 mode at 0.5 MHz. Using the *m* parameter values in Figure 3, we attempted to re-scale the peak values using the scaling value,

(24) $$R=\frac{{m|}_{A0}}{{m|}_{S0}}.$$

The calculated results of scaling the peak values are shown in Table 1. Using *R* seemed to be a reasonable way to correct for the acoustic leakage’s mode dependence since it reduced the percent difference to 10.0%.

However, when we attempted to use *R* to scale the air-coupled spectra in the same way, we did not get the PVDF spectra. In Figure 9b, the spectra between phase velocities of 3000 and 10,000 m/s were scaled using R and then Figure 9a,b spectra were normalized to facilitate comparison. This type of implementation of the scaling increased the noise as well as the features of the spectra, and it did not account for how the variance should change when features become more or less prominent. This is most evident in Figure 9b where the S0 mode is spread out, noisy, and seems to lack a distinctive peak as in Figure 9a. The noisiness and lack of a distinctive peak, in particular, seemed to greatly hinder the quality of the scaling. The lack of a distinctive peak was most likely due to the previously discussed variation shown in Figure 5.

**Figure 10:**
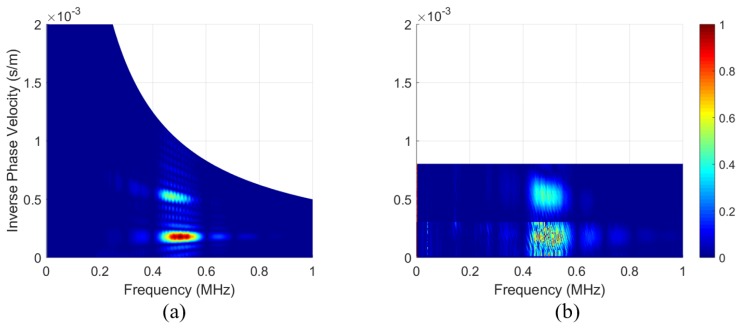
(**a**) Normalized inverse phase velocity–frequency spectrum that was measured using the PVDF transducer; and (**b**) rescaled, then normalized inverse phase velocity–spectrum that was measured using the air-coupled transducer.

As mentioned previously (see Equation (1)), the phase velocity is inversely proportional to the sine of the air-coupled transducer’s angular position. Since this leads to a loss in resolution at higher phase velocities, it was found that the features of the spectra are easier to compare using the inverse phase velocity (or slowness), as shown in Figure 10. This figure also excludes the dispersion curves and focuses on a smaller frequency range to aid in the comparison.

## 5. Conclusions

In this paper, we demonstrated an alternate method for measuring a wavenumber spectrum using an air-coupled transducer. The simple approach leverages Snell’s law to relate the angular position of the air-coupled transducer to the wavenumber spectrum of a Lamb wave. Consequently, this also demonstrates the extent of the air-coupled transducer’s angular sensitivity. The method detailed in this paper reduces the amount of computation required to measure a wavenumber spectrum, and allows for a wavenumber spectrum measurement without having to physically scan more than one point on the sample. The latter benefit is ideal for small samples or for measurements in cramped spaces.

However, the method does have several drawbacks. The most significant drawback is the acoustic leakage’s mode dependence that inherently biases the spectra measured by the air-coupled transducer. Another drawback is that the method requires very small angular increments to accurately measure Lamb waves at higher phase velocities. We addressed the acoustic leakage’s mode dependence by proposing a scaling method that uses a ratio of acoustic leakage amplitude to wave-structure amplitude. It should be noted that this mode dependence would still be a problem if one were to measure spectra by using the conventional 2DFFT approach with an air-coupled transducer.

Potential future work could include: refining the scaling method, limiting the directivity of the air-coupled transducer to just waves that are perpendicular to the transducer face, and using a more accurate angular positioning system with the ability to use smaller increments. The resolution at higher phase velocities could also be improved by using water instead of air because the higher wave speed in water will increase the angle of incidence at which a given phase velocity can be measured. Since an angle of incidence of 0° corresponds with $c_{p}\approx \infty$, the first few degrees represent a large range of phase velocities. Increasing the angle of incidence at which these phase velocities can be measured would effectively shift phase velocities away from these content-rich angles.

## Figures and Tables

**Table 1:** Calculated results for scaling the peak values of the air-coupled spectra to approximate the PVDF spectra.

	$\frac{\max\left(S0\;\mathrm{spectra}\right)}{\max\left(A0\;\mathrm{spectra}\right)}$	$R=\frac{{m|}_{A0}}{{m|}_{S0}}$	Scaled Peak Values for Comparison	% Difference
PVDF	1.8613	N/A	1.8613	
air-coupled	0.4067	5.0556	2.0561	10.0%

## Nomenclature

$\theta_{n}$	Plane wave propagation angle in media *n* with respect to the surface’s normal
$\sigma_{ij}$	2nd-order stress tensor
ρ	Mass per unit volume of an elastic solid
$u_{i}$	Particle displacement vector
$C_{ijkl}$	4th-order stiffness tensor of an elastic solid
$\epsilon_{kl}$	2nd-order strain tensor
$k_{j}$	Wavevector
${\hat{k}}_{j}$	Normalized wavevector
$r_{j}$	Spatial coordinate vector
ω	Frequency in terms of radians/second
$c_{L}$	Longitudinal wave speed in an elastic solid
$c_{S}$	Shear wave speed in an elastic solid
$\rho_{o}$	Ambient mass per unit volume in air
$\rho^{\prime }$	Change in mass per unit volume in air
$p^{\prime }$	Change in pressure in air
$c_{air}$	Wave speed in air
$v_{air}^{\prime }$	Change in the particle velocity in air
$B^{\left(air\right)}$	Acoustic leakage’s particle displacement amplitude
*H*	Plate waveguide thickness
i,j,k,l	Indices such that: *i,j,k,l = x,y,z*
